# Material Hardship, Mental Health, and Parenting Stress among Grandparent Kinship Providers in COVID-19

**DOI:** 10.1093/geroni/igaa057.3483

**Published:** 2020-12-16

**Authors:** Yanfeng Xu, Qi Wu, Sue Levkoff, Merav Jedwab

**Affiliations:** 1 University of South Carolina, Columbia, South Carolina, United States; 2 Arizona State University, Phoenix, Arizona, United States; 3 Hadassah Academic College, Jerusalem Israel, Israel

## Abstract

The COVID 19 pandemic has exposed the vulnerability of many families, including grandparent kinship families, to deal with a health/economic crisis. The fear of COVID-19 plus stay-at-home orders have increased individuals’ psychological distress. Moreover, school closures and homeschooling further increased parenting stress among caregivers. This study examined the relationship between material hardship and parenting stress among grandparent kinship providers and assessed grandparents’ mental health as a potential mediator to this relationship during the COVID-19 pandemic in the United States. Grandparent kinship providers (N=362) that took primary care of their grandchildren participated in a cross-sectional survey via Qualtrics Panels in June 2020 in the United States. Descriptive and bivariate analyses, binary logistic regression, and mediation analyses were conducted using STATA 15.0. Experiencing material hardship (OR = 1.67, p < 0.001) was significantly associated with higher odds of parenting stress among grandparent kinship providers, and grandparents’ mental health (indirect effect = 0.11, 95% CI [0.01, 0.25]) partially mediated this association. Addressing material and mental health needs among grandparent kinship providers is critical to decreasing their parenting stress.

